# Influence of morphology and hemodynamic factors on rupture of multiple intracranial aneurysms: matched-pairs of ruptured-unruptured aneurysms located unilaterally on the anterior circulation

**DOI:** 10.1186/s12883-014-0253-5

**Published:** 2014-12-31

**Authors:** Ying Zhang, Xinjian Yang, Yang Wang, Jian Liu, Chuanhui Li, Linkai Jing, Shengzhang Wang, Haiyun Li

**Affiliations:** Department of Interventional Neuroradiology, Beijing Neurosurgical Institute, Beijing Tiantan Hospital, Capital Medical University, Beijing, China; Department of Mechanics and Engineering Science, Fudan University, Shanghai, China; Department of Biomedical Engineering, Capital Medical University, Beijing, China

**Keywords:** Intracranial aneurysms, Multiple aneurysm, Anterior circulation, Rupture, Computational fluid dynamics

## Abstract

**Background:**

The authors evaluated the impact of morphological and hemodynamic factors on the rupture of matched-pairs of ruptured-unruptured intracranial aneurysms on one patient’s ipsilateral anterior circulation with 3D reconstruction model and computational fluid dynamic method simulation.

**Methods:**

20 patients with intracranial aneurysms pairs on the same-side of anterior circulation but with different rupture status were retrospectively collected. Each pair was divided into ruptured-unruptured group. Patient-specific models based on their 3D-DSA images were constructed and analyzed. The relative locations, morphologic and hemodynamic factors of these two groups were compared.

**Results:**

There was no significant difference in the relative bleeding location. The morphological factors analysis found that the ruptured aneurysms more often had irregular shape and had significantly higher maximum height and aspect ratio. The hemodynamic factors analysis found lower minimum wall shear stress (WSSmin) and more low-wall shear stress-area (LSA) in the ruptured aneurysms than that of the unruptured ones. The ruptured aneurysms more often had WSSmin on the dome.

**Conclusions:**

Intracranial aneurysms pairs with different rupture status on unilateral side of anterior circulation may be a good disease model to investigate possible characteristics linked to rupture independent of patient characteristics. Irregular shape, larger size, higher aspect ratio, lower WSSmin and more LSA may indicate a higher risk for their rupture.

## Background

Approximately 15%-35% of aneurysm patients have multiple aneurysms [1-4]. Neurosurgeons face a special problem to treat them because when patient’s general clinical condition can’t undergo a full treatment or aneurysms are presented in locations that cannot be operated through a single treatment, the high-risk or duty aneurysm should be identified and operated first [5,6]. For patients with subarachnoid hemorrhage (SAH), combing clinical, CT and angiographic findings, most bleeding sites can be identified but there are still some difficult cases. The misjudgment is dangerous because the untreated but true ruptured aneurysm may bleed again very soon. As for patients without SAH, considering the high rates of morbidity and mortality in the event of rupture, discriminating the aneurysms in danger of rupture before operation is valuable for preventive treatment too.

Previous studies displayed a number of characteristics that may be related to ruptured aneurysms, such as size, location, irregular shape (i.e. blebs, nipples or multiple lobes), etc. [7-9]. And recently, computational fluid dynamics (CFD) has become a popular tool for studying intracranial aneurysm hemodynamics and discriminating rupture status [10].

In this study, we investigated the rupture-related characteristics on 20 matched-pairs of ruptured-unruptured saccular aneurysms located unilaterally on the anterior circulation in the same patient. For they provide an ideal internal control for such patient-specific variables as genetic, age, sex, blood pressure, individual habits (including smoking, alcoholism), comorbidities present and so forth, and only focus on aneurysms characteristics. We compared their sites, morphology and hemodynamic factors and hope to get some illuminating results on aneurismal rupture. To our knowledge, the matched rupture risk data in one patient including morphological and hemodynamic factors is rarely reported.

## Methods

### Source of patients

**Figure 1:**
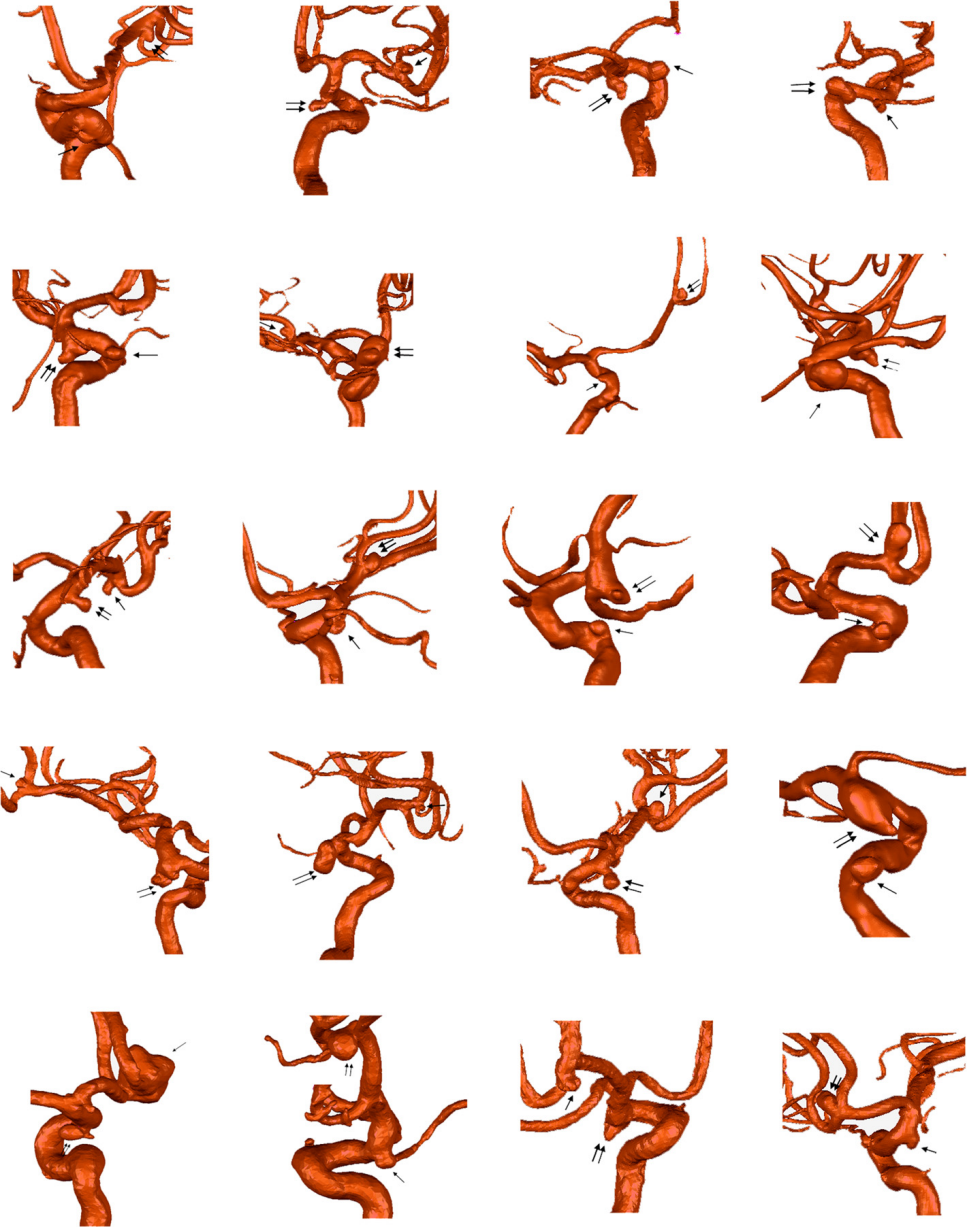
**Visualization of 20 multiple aneurysms pairs.** Double arrows show the ruptured aneurysms while single arrows show the unruptured aneurysms.

From January 2012 to December 2013, a total of 20 patients with ruptured-unruptured aneurysms pairs in ipsilateral anterior circulation were selected from our institution’s data base (Figure 1).

The inclusion criteria were: (1) 2 saccular aneurysms with different rupture status in one patient; (2) in ipsilateral anterior circulation. The specific locations include internal carotid artery (ICA, the Bouthillier classification: C4, cavernous; C5, clinoid; C6, ophthalmic; C7, communicating), anterior cerebral artery (ACA), middle cerebral artery (MCA), and anterior communicating artery (ACoA) with inflow only from the ipsilateral ACA (to exclude the contralateral carotid system’s effect); (3) first SAH; (4) complete clinical data and three-dimensional (3D) DSA images were of adequate resolution for CFD analysis; (5) the rupture site was identified by head CT scan imaging or intraoperative findings; (6) the study was consented to by patients or their close relatives. The exclusion criteria were: (1) fusiform or dissecting aneurysms; (2) ACoA aneurysms with good inflow from both sides of ACA; (3) previous SAH; (4) incomplete clinical data or 3D-DSA images were not of adequate resolution for CFD analysis; (5) the rupture site cannot be identified; (6) patients and their relatives did not consent to the study.

All of them were emergency patients for cerebral hemorrhage, underwent DSA at the first time within 2 days before vasospasm happened in our hospital. Written informed consents were obtained from all patients, and the research was permitted by the ethics committee of Beijing Tiantan Hospital. Each pair was divided into two groups, ruptured and unruptured.

### Modeling of aneurysms and numerical simulation

Numerical simulation of blood hemodynamics was performed as described previously [11-14]. Briefly, patient-specific 3D-DSA data of all aneurysm pairs, with each case sharing inlet on the same ICA cervical segment, were obtained before treatment. In each case, the last aneurysm along with the brain blood flow was defined as the distal aneurysm, the other aneurysms was defined as the proximal aneurysm. Software package developed in-house was used to create and modify a stereolithographic image that contained the blood vessel luminal surface information. Each model was imported into the ICEM CFD software (ANSYS, Canonsburg, Pennsylvania, USA) to create more than one million finite volume tetrahedral element grids. After meshing, ANSYS CFX 12.1 software was used for simulation of blood hemodynamics. The inflow boundary condition was a pulsatile period velocity profile that was obtained by transcranial Doppler. Two cardiac cycle simulations were performed for numerical stability. The results from the second cardiac cycle were collected as output for the final analyses. The average Reynolds number was within the range of normal blood flow in human cerebral arteries.

### Data analysis

Morphologic parameters including the aneurismal size (maximum height), neck width and aspect ratio (dome-to-neck ratio) were measured and calculated from 3D-DSA data. The number of each morphologic types (lateral/bifurcation, regular/irregular) and relative bleeding location (distal/proximal) were recorded. After CFD simulation, these flow pattern characteristics in the aneurysms were analyzed: 1) flow stability, the stable flow pattern persisted during the cardiac cycle, while the unstable flow had flow structure moved or changed during the cardiac cycle [15]; 2) flow complexity, the simple flow pattern had a single vortex structure, while the complex flow contained multiple vortices [15]. The following wall shear stress (WSS) related quantitative parameters were calculated: maximum and minimum WSS (WSSmax, WSSmin), time-averaged WSS (TAWSS), oscillatory shear index (OSI) and low WSS area (LSA). LSA was defined as the proportion of the low WSS area (below 10% of the mean WSS at the anterior genu of the carotid siphon) to the whole area of the aneurysm [16]. All the parameters in the ruptured and unruptured group were calculated and compared.

For qualitative data of relative bleeding location (distal/proximal), morphology type (lateral/bifurcation, regular/irregular), flow stability and complexity, the location of WSSmax (neck/dome) and WSSmin (neck/dome), McNemar’s test was used to compare the differences between the two groups. For quantitative data, one-sample Kolmogorov–Smirnov test was used to test the normal distribution. Then paired-sample t test was used for all the approximately normally distributed parameters with data expressed as mean (SD), and Wilcoxon’s sign rank test was used for non-normally distributed parameters with data expressed as median (quartile). *p* < 0.05 was regarded as statistically significant. Statistical analysis was performed with an SPSS 15.0 package.

## Results

### Characteristics of patients and aneurysms

Table 1 shows clinical characteristics of these 20 patients. There were 2 male and 18 females aged between 39 and 83 years (mean 60.6y). For health habits, 4 (20%) were alcohol drinkers, 3 (15%) were cigarette smokers. For comorbidity, 3 (65%) had hypertension (HBP), 6 (30%) had deep vein thrombosis (DVT), 5 (25%) had hyperlipidemia (HPL), 4 (20%) had hyperhomocysteinemia (HHCY), atherosclerosis (AS), 2 (10%) had diabetes mellitus (DM), coronary heart disease (CHD), and 1 (5%) had brain hernia (BH), polycystic kidney disease (PKD), cerebral infarction (CI). 7 (35%) patients had other coexisting aneurysms on the contralateral side of anterior circulation or vertebrobasilar artery. Patients were graded according to the Hunt and Hess scale: grade 1 or 2: 65% and grade ≥3: 35%. For the treatment, 16 (80%) were partial treatments (i.e., some coexisting unruptured aneurysms were left untreated), 12 of them were only rupture-site aneurysm coiling/clipping and 4 of them were only aneurysms on rupture-side clipping; 2 (10%) were two-stage treatments (i.e., complete treatment through 2 operations), one of them was clipping following rupture-site coiling, the other of them was coiling following rupture-side clipping; 2 (10%) chose no operation.

**Table 1:** **Clinical characteristics of these 20 patients**

**No.**	**Habit, S/D**	**Comorbidity**	**Site** ^**†**^	**Other site#**	**Hunt-hess grade**	**Operation**
1		HBP, BH	MCA*, C5		4	Bled-site clipping
2			C7*, MCA		1	Bled-site coiling
3		HBP, AS, DVT	C7*, C6		2	Bled-site coiling
4		HPL, PKD	C6* , C7		2	Bled-site coiling
5	D	DM, HPL, CI, DVT	C7*, C6		2	Bled-site clipping
6			C6*, MCA		2	Bled-site coiling
7	D	HBP	ACA*, C5		2	Bled-site coiling
8	S	HHCY	C7*, C6		2	Bled-site clipping
9		HBP	C7*, MCA	ACA	2	Two-stage treatment
10		HBP	MCA*, C7		3	Bled-site coiling
11			C7*, C4	C4	1	No operation
12	S,D	HBP, DVT	ACoA*, C6		3	Bled-site coiling
13	S	HBP, HPL,HHCY, AS, DVT,	C7*, MCA		3	Bled-site coiling
14		HBP, HPL, CHD	C7*, MCA	C7	2	Bled-site coiling
15		HBP, DM, AS	C7*, MCA	C7	2	Bled-side clipping
16		HBP, HHCY, HD	C7*, C4	C7, C4	3	No operation
17		HBP, HPL	C7*, ACoA		3	Bled-side clipping
18		HBP	MCA*, C7	C7	3	Two-stage treatment
19		HBP, DVT	C7*, MCA		1	Bled-side clipping
20	D	HHCY, AS, DVT	ACoA*, C7	BA	1	Bled-side clipping

S/D, Smoker/drinker; HBP, Hypertension; BH, Brain hernia; AS, Atherosclerosis ; DVT, Deep vein thrombosis; HPL, Hyperlipidemia; PKD, Polycystic kidney disease; DM, Diabetes mellitus; CI, Cerebral infarction; HHCY, Hyperhomocysteinemia; CHD, Coronary heart disease;

^†^the Bouthillier classification of internal carotid artery (ICA) segments: C4, Cavernous; C5, Clinoid; C6, Ophthalmic; C7, Communicating; MCA, Middle cerebral artery; ACA, Anterior cerebral artery; ACoA, Anterior communicating artery;

#coexisting aneurysms on the contralateral side of anterior circulation or vertebrobasilar artery; BA, basilar artery.

*the ruptured group.

The relative rupture location were 11 (55%) distal aneurysms and 9 (45%) proximal aneurysms. Of the ruptured aneurysms 12 were on C7, 3 on MCA, 2 on C6, ACoA and 1 on ACA; of the unruptured aneurysms 7 were on MCA, 4 on C7, C6, 2 on C5, C4, and 1 on ACoA. There was no significant difference in the relative bleeding location (distal/proximal) between the ruptured and unruptured group (Table 2).

**Table 2:** **Relative locations and morphologic factors**

**Variables**	**Aneurysms**			***p*** **value ‡ (2-tailed)**
	**Total (n = 40)**	**Ruptured group (n = 20)**	**Unruptured group (n = 20)**	
Relative location				
Distal (%)	20 (50)	11 (55)	9 (45)	0.824
Proximal (%)	20 (50)	9 (45)	11 (55)	
Morphology type				
Lateral (%)	15 (37.5)	6 (30)	9 (45)	0.581
Bifurcation (%)	25 (62.5)	14 (70)	11 (55)	
Regular	22 (55)	5 (25)	17 (85)	0.002
Irregular	18 (45)	15 (75)	3 (15)	
Maximum height, mm	3.94 (1.977)	4.68 (1.605)	3.20 (2.076)	0.041
Neck width, mm	3.58 (1.308)	3.77 (1.468)	3.39 (1.131)	0.351
Surface area, mm^2^	32.20 (38.809)	42.82 (41.895)	27.14 (15.980)	0.062
Volume,mm^3^	20.85 (41.064)	31.18 (43.944)	16.08 (14.697)	0.108
Aspect ratio	1.11 (0.399)	1.31 (0.402)	0.92 (0.290)	0.004

The comparisons of maximum height and aspect ratio between the two groups were of significant differences (*p* < 0.05). (‡, McNemar’s test, paired-sample t test or Wilcoxon’s sign rank test as appropriate).

### Comparison of morphologic factors between ruptured and unruptured group

For aneurysm morphology types, the ruptured aneurysms were more common irregular type, but there were no significant differences in the lateral/bifurcation type between the ruptured and unruptured groups.

As presented in Table 2, the ruptured aneurysms had significantly higher maximum height and aspect ratio than the unruptured aneurysms. But there were no significant differences in the neck width, surface area or volume between the ruptured and unruptured group.

### Comparison of hemodynamic factors between ruptured and unruptured group

**Figure 2:**
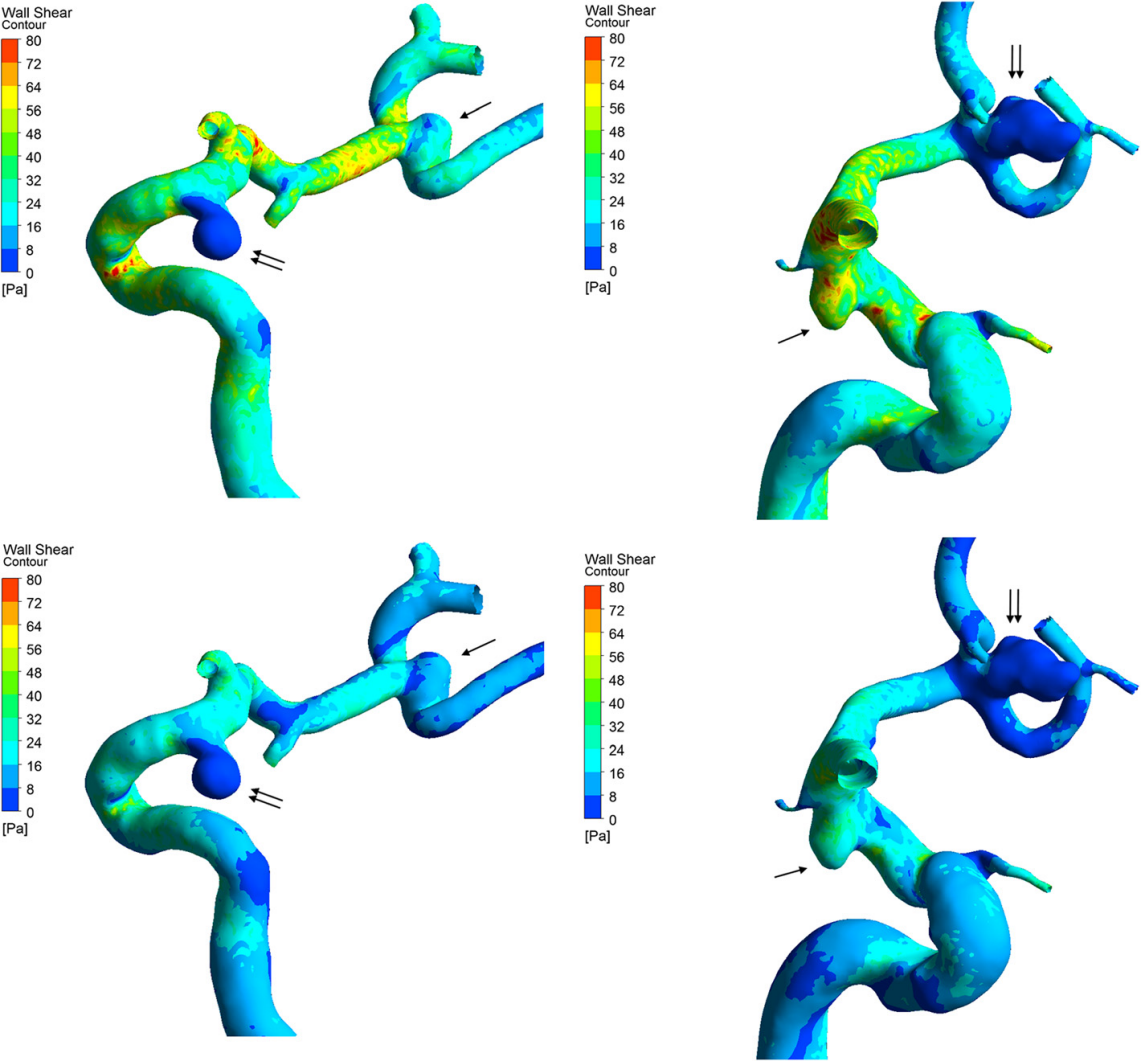
**Visualization of the wall shear stress (WSS) of 2 representative aneurysms pairs (case 1, left; case 2, right; upper, at the systolic peak; lower, at the diastolic end).** Double arrows show the ruptured aneurysms while single arrows show the unruptured aneurysms. Case 1 has aneurysms on ICA C7 (duty site) and MCA. Case 2 has aneurysms on ICA C7 and ACoA (duty site). The ruptured aneurysms have lower WSS on the dome and more low WSS area.

As presented in Table 3 and Figure 2, the ruptured aneurysms had significantly lower WSSmin and more LSA than the unruptured aneurysms. The ruptured aneurysms more often had WSSmin on the dome. Other hemodynamic factors showed no significant differences between the two groups.

**Table 3:** **Hemodynamic factors**

**Variables**	**Aneurysms**			***p*** **value‡ (2-tailed)**
	**Total (n = 40)**	**Ruptured group (n = 20)**	**Unruptured group (n = 20)**	
WSSmax, Pa	53.72 (26.075)	55.65 (32.700)	51.78 (17.845)	0.607
WSSmin, Pa	0.75 (1.268)	0.28 (0.289)	1.22 (1.658)	0.020
TAWSS, Pa	13.87 (8.774)	12.72 (10.234)	15.02 (7.105)	0.392
OSI, Pa	0.0090 (0.00940)	0.0104 (0.02054)	0.0061 (0.00871)	0.156
LSA,%	0.40 (4.344)	2.55 (16.536)	0.12 (1.044)	0.011
Flow stability				
Stable (%)	14 (35)	6 (30)	8 (40)	0.754
Unstable (%)	26 (65)	14 (70)	12 (60)	
Flow complexity				
Simple (%)	11 (27.5)	3 (15)	8 (40)	0.125
Complex (%)	29 (72.5)	17 (85)	12 (60)	
WSSmax location				
Neck (%)	33 (82.5)	17 (85)	16 (80)	1.000
Dome (%)	7 (17.5)	3 (15)	4 (20)	
WSSmin location				
Neck (%)	9 (22.5)	1 (5)	8 (40)	0.039
Dome (%)	31 (77.5)	19 (95)	12 (60)	

The comparisons of WSSmin, LSA and WSSmin location on the aneurysms between the two groups were of significant differences (*p* < 0.05). (‡, paired-sample t test, Wilcoxon’s sign rank test or McNemar’s test as appropriate).

## Discussion

Multiple aneurysms locating unilaterally on the anterior circulation are common and propose special challenge for neurosurgery. K. Mizoi *et al*. [17] mentioned that 19.4% (72/372) multiple aneurysms located unilaterally in the anterior circulation (not including the ACoA aneurysm) and 42.2% (157/372) multiple aneurysms included at least one on the ACoA (whether they located unilaterally or bilaterally was not concerned). For surgical outcomes, 17 of them got partial treatments only and of which 1 patient died due to postoperative bleeding from the untreated aneurysms [17]. Y. Orz. *et al*. [18] reported that 66.5% (147/221) multiple aneurysms located unilaterally in the anterior circulation, the total mortality rate after surgery is 15.2% and 15 of them got partial/two-stage treatments [18]. For Rinne *et al*. [19], the incidence percentage was 16.2% (49/302) and their results suggested that multiple aneurysms signicifantly increase the risk for poor outcome. For Imhof *et al*. [20], 44.6% (54/121) ruptured aneurysms had the coexisting unruptured aneurysms ipsilateral to the ruptured one. Of all the 124 patients with SAH and multiple aneurysm, 3 passed away immediately after admission and 64 got partial/two-stage treatments [20]. Partial/two-stage treatments are not rare for multiple aneurysms and it put forward a need to identify risky ones among all the coexisting aneurysms. Ruptured-unruptured aneurysm pair on the same patient’s ipsilateral anterior circulation is very interesting. It may be a good disease model not only to investigate characteristics linked to bled aneurysms but also to understand multiple aneurysms better.

As regards to the clinical data, we demonstrate that most cases are female (consistent with previous reports for association between the presence of multiple aneurysms and female sex [1,3,4]) and 65% have hypertension. Other coexisting additional disorders, such as DVT, HPL, AS, etc., can also exist. However, the precise role of these clinical factors in assessing rupture risk needs further study with larger sample size and comparative control group. There is no difference for which relative location (proximal/distal) would bleed first, so Liang-Der Jou *et al*.’s [21] previous speculation that the proximal aneurysm in serial aneurysms may be subject to a greater rupture risk whose research based on only hemodynamic analysis on 4 multiple aneurysms pairs but without clinical data analysis was not true situation for patients.

The ruptured aneurysms are more common to have irregular shape. Some clinical experience and human studies have reported the same found [7,13,22-24]. The focal bulges on the surface of aneurysm often indicates a weak and thinner wall which indicates a higher risk of aneurysmal rupture [13,24].

The natural history studies on unruptured aneurysms (including single aneurysms and multiple aneurysms together) proposed aneurysms size to be key predictor for rupture, and diameters > 7 mm/10 mm represented much higher bleeding risk [7,25-27]. In our study, the ruptured aneurysms have higher maximum height too. But studies on only multiple aneurysms seem to have different results. In our study the mean maximum height for all these 40 aneurysms is 3.94 ± 1.977 mm, which means that most aneurysms are small aneurysms with diameter <7 mm in size. Similarly, Jagadeesan *et al*. [28] reported that very small(≤3 mm) and small aneurysm (>3 mm but ≤7 mm) constituted the majority of ruptured aneurysms (24.6% and 50.7%) for multiple aneurysms. Lu *et al*.’s [2] retrospective study of 294 multiple aneurysms reported that 88.1% of the aneurysms were 5 mm or less. Size may be a good assessment factor for multiple aneurismal rupture, and what is interesting, multiple aneurysms maybe have a smaller size. However, further multicenter studies on larger multiple aneurysms databases are needed to verify the true situation.

The ruptured aneurysms have higher aspect ratio. Consistent with our observation, Sadatomo *et al.* [29] found that in cases with aspect ratio more than or equal to 1.8, 87% were ruptured aneurysms, whereas in cases with aspect ratio less than 1.8, 92% were unruptured aneurysms. Backes *et al.* [8] performed conditional univariable logistic regression analysis on 124 patients with 302 multiple aneurysms and found that aspect ratio ≥1.3 was associated with multiple aneurysm rupture independent of aneurysm size and location, and independent of patient characteristics. Ujiie *et al.* [30] performed an in vivo study and found that aspect ratio determined the intra-aneurysmal flow, a higher aspect ratio (>1.6) indicated a much slower circulation near the dome and flow stagnation was detected in the dome of irregular-shaped aneurysms (dumbbell-shaped and bleb) [30]. The dome often has flow stagnation [31,32], which is related to intra-aneurysmal thrombosis and subsequent inflammatory changes in the aneurysm wall [30]. Higher aspect ratio may suggest a remodeling artery wall and risk for aneurismal rupture.

For hemodynamic factors analysis, we find that the ruptured aneurysms have lower WSSmin and more LSA. These data are consistent with our previous results showing that low WSS might be involved in increasing the risk of rupture [13]. The ruptured aneurysms more often have WSSmin on the dome. Previous studies found that unruptured aneurysms’ thin-walled dome regions co-localized with low WSS and the ruptured aneurysms’ bleeding points were often located at the dome with a low WSS [33]. So we suspect that WSSmin may be associated with flow stagnation at the dome as well as the localized pathologic aneurysm wall degeneration and thinning on the dome. These hemodynamic factors may help us to verify ruptured aneurysms and offer some insight into rupture mechanisms.

The present study had some limitations that need to be addressed. Rigid wall, laminar flow and Newtonian blood were used in our present aneurysm models. The flow conditions were not patient-specific. It is reported that using normal-resolution meshes (1 to 5 million tetrahedral) may underestimate the aneurysm WSS and WSSmax when compared with using high-resolution meshes (300,000 to 1 million elements) [10], while using generalized (typical flow rates in a healthy adult) and patient-specific inflow boundary conditions may results in different WSS magnitudes and hemodynamic characteristics [34]. However, matched pairs design in our study may help control for individual differences affecting the results. Sharing the same inflow boundary conditions on one side of ICA especially help avoid inflow boundary condition confounding. The other limitations were the effects of small sample size and a single center selection bias.

## Conclusions

Intracranial aneurysms pairs with different rupture status in one patient’s ipsilateral anterior circulation may be a good disease model to investigate possible features linked to bled aneurysms independent of patient characteristics. The ruptured aneurysms manifested irregular shape, larger size, higher aspect ratio, lower WSSmin and more LSA compared with their unruptured mates. It may promote a better understanding of multiple aneurysms and help neurosurgeons to identify risky sites before operation.

## Abbreviations

ACA: Anterior cerebral artery
ACoA: Anterior communicating artery
AS: Atherosclerosis
BH: Brain hernia
CFD: Computational fluid dynamics
CHD: Coronary heart disease
CI: Cerebral infarction
DM: Diabetes mellitus
DVT: Deep vein thrombosis
HBP: Hypertension
HHCY: Hyperhomocysteinemia
HPL: Hyperlipidemia
ICA: Internal carotid artery
LSA: Low wall shear stress area
MCA: Middle cerebral artery
OSI: Oscillatory shear index
PKD: Polycystic kidney disease
TAWSS: Time averaged wall shear stress
WSS: Wall shear stress
WSSmax: The maximum wall shear stress
WSSmin: The minimum wall shear stress
